# Cancer Predisposition Syndromes Associated With Pediatric High-Grade Gliomas

**DOI:** 10.3389/fped.2020.561487

**Published:** 2020-11-12

**Authors:** Giulia Ceglie, Giada Del Baldo, Emanuele Agolini, Martina Rinelli, Antonella Cacchione, Francesca Del Bufalo, Maria Vinci, Roberto Carta, Luigi Boccuto, Evelina Miele, Angela Mastronuzzi, Franco Locatelli, Andrea Carai

**Affiliations:** ^1^Department of Pediatric Hematology/Oncology and Cell and Gene Therapy, Istituto di Ricovero e Cura a Carattere Scientifico Bambino Gesù Children's Hospital, Rome, Italy; ^2^Laboratory of Medical Genetics, Istituto di Ricovero e Cura a Carattere Scientifico Bambino Gesù Children's Hospital, Rome, Italy; ^3^Greenwood Genetic Center, Greenwood, SC, United States; ^4^Clemson University School of Health Research, Clemson, SC, United States; ^5^Sapienza, University of Rome, Rome, Italy; ^6^Neurosurgery Unit, Department of Neurological and Psychiatric Sciences, Istituto di Ricovero e Cura a Carattere Scientifico Bambino Gesù Children's Hospital, Rome, Italy

**Keywords:** brain tumors, cancer predisposition, genetics of cancer, pediatric neuro- oncology, high grade gliomas

## Abstract

Pediatric High-Grade Gliomas (pHGG) are among the deadliest childhood brain tumors and can be associated with an underlying cancer predisposing syndrome. The thorough understanding of these syndromes can aid the clinician in their prompt recognition, leading to an informed genetic counseling for families and to a wider understanding of a specific genetic landscape of the tumor for target therapies. In this review, we summarize the main pHGG-associated cancer predisposing conditions, providing a guide for suspecting these syndromes and referring for genetic counseling.

## Introduction

Central Nervous System (CNS) tumors are the most common pediatric solid tumors and represent the second most frequent neoplasm in pediatric age, second only to leukemias. They count for 1.12–5.14 cases per 100,000 people in individuals aged 0–19 years, with variable incidence rates across different countries, the highest being in the USA (1). Management of pediatric CNS tumors is challenging and requires specific oncological training.

Among brain tumors in the pediatric age, gliomas are the most represented. Approximately 21% of all primary pediatric gliomas are high-grade tumors (2, 3). Even though from a histopathological point of view pediatric high-grade gliomas (pHGGs) are similar to their adult counterpart, their genetic and epigenetic features reflect intrinsic differences compared to adult HGGs. Despite an increased understanding of their biological basis, therapeutic options for these tumors are still very limited, and the long-term prognosis remains poor, with high levels of both morbidity and mortality (3, 4) and a 5-year survival rate of <20% (4).

Risk factors for pHGG seem to be mostly genetic in nature, even though some predisposing environmental factors such as irradiation have been described (5). In contrast to adult population, where cancer associated mutations are mostly somatic and resulting from external causes, germline mutations are frequently encountered in children.

Several cancer predisposing syndromes (CPS) associated with an increased risk of developing to pHGG have been identified so far, including Neurofibromatosis type 1 (NF1), Turcot syndrome and Li-Fraumeni syndrome. In this review we will address the impact of these syndromes for the management of pHGG.

## Methods

The authors conducted a literature search describing CNS tumors and cancer predisposing syndromes. Selection of studies were based on research topics (such as cancer predisposition syndrome AND/OR brain tumor genetics, brain tumor cancer predisposition syndrome, HGG predisposition syndromes, HGG in childhood) found in the PubMed. Only papers written in the English language and those published from the year 2000 up to May 2020 were selected. We included reviews, case series and research studies that were classified according to their relevance. No abstracts were included. The information found in the selected studies was carefully evaluated, which is described and discussed in the following sections.

## LI-Fraumeni Syndrome

Li-Fraumeni Syndrome (LFS) (OMIM #151623) was reported for the first time in 1969 by Frederick Li and Joseph Fraumeni (6). LFS is an autosomal dominant, highly penetrant cancer predisposition syndrome associated with germline mutations in the *TP53* gene. It lacks additional clinical features and is only characterized by the high frequency of malignancies in multiple organs, making it a difficult syndrome to diagnose in the absence of a significant family history of multiple cancers (7). The involved gene encodes the *TP53* transcription factor, tumor protein p53, also known as the “guardian of the genome” (8). *TP53* is involved in cellular growth control by regulating the expression of several genes causing cell-cycle arrest and apoptosis in response to DNA damage.

### Epidemiology and Cancer Spectrum

LFS prevalence is estimated between 1/5.000 and 1/20.000 of the population (9, 10), even if the estimated prevalence of pathogenic and likely pathogenic germline *TP53* variants seems to be higher, as described by Andrade et al. (9).

LFS is characterized by a high lifetime cancer risk and, due to its extremely high penetrance, by a familiar clustering of tumors. Cancer types are variable and often present during childhood. Osteosarcoma, soft-tissue sarcomas, brain tumors, early-onset breast cancer, leukemia, and adrenocortical tumors are the most frequently observed tumors (10). It can also be associated with myelodysplastic syndromes, lymphoma and other benign and malignant tumors (11, 12). In children with LFS, brain tumors are the second most common malignancies following adrenocortical carcinoma. A quarter of childhood tumors involved CNS compared to only 13% of adult LFS related tumors (13). In LFS, the median age of onset of brain tumors is 16 years, compared to 57 years in the general population.

CNS tumors related to LFS have a prevalence ranging from 9 to 14% (14) and the most frequent types are glioblastoma and astrocytoma. Nonetheless, medulloblastoma, ependymoma, choroid plexus carcinomas, and other embryonal tumors are also described.

### Etiopathology

**Figure 1:**
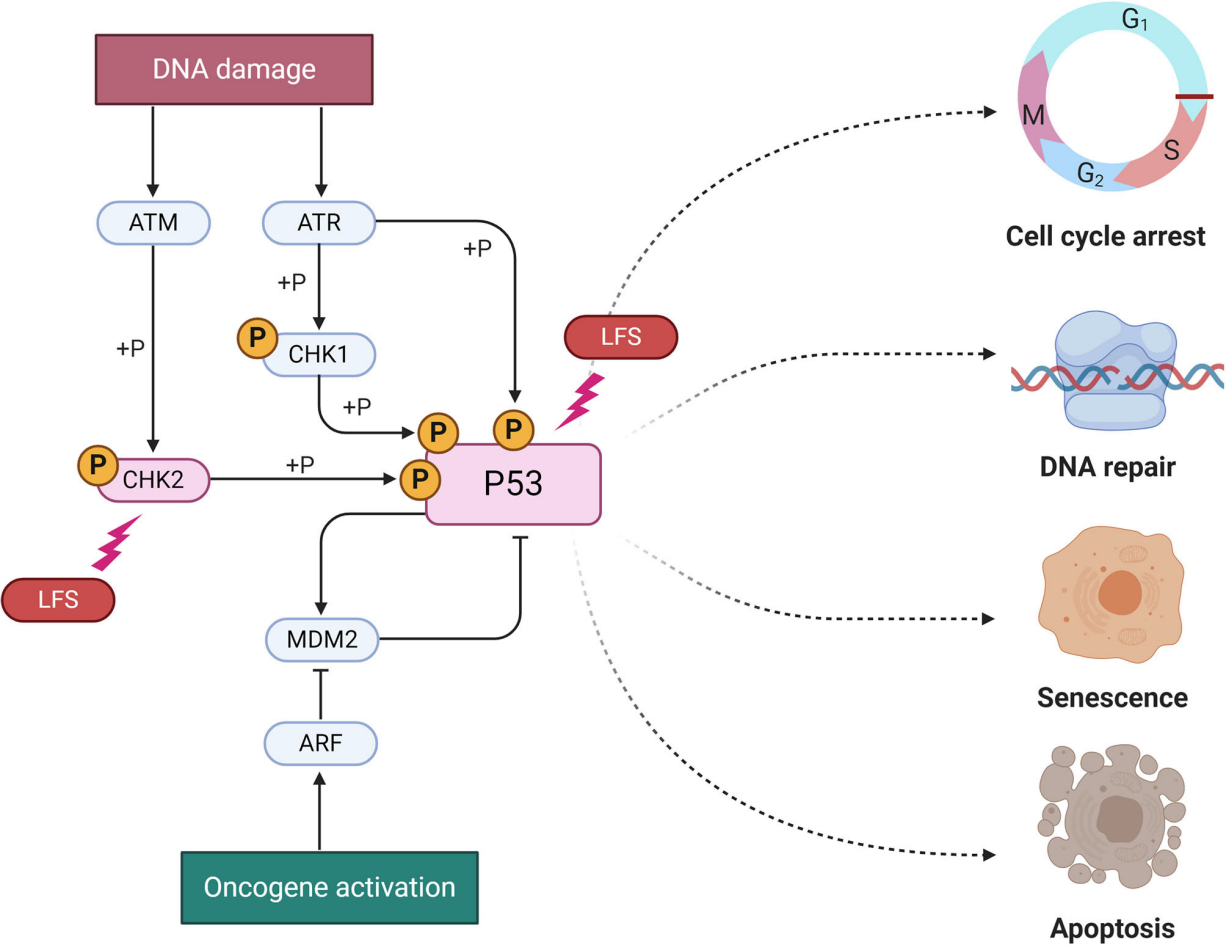
Molecular pathways of Li-Fraumeni (LFS) Syndrome. The two known mutation for LFS are represented here (*P53* and *CHK2*) as lighting bolt. A group of protein kinases such as ATM, ATR, CHK1, CHK2 is implicated in the genome integrity checkpoint, a molecular cascade that detects and responds to several forms of DNA damage caused by genotoxic stress. Oncogenes also stimulate p53 activation, mediated by the protein ARF. In a normal cell, p53 is inactivated by its negative regulator, *MDM2*. Upon oncogene activation, various pathways will lead to the dissociation of the *P53* and *MDM2* complex. Once activated, p53 will induce a cell cycle arrest to allow either repair and survival of the cell or apoptosis to discard the damaged cell. Adapted from “P53 Regulation and Signaling,” by BioRender.com (2020). Available online at: https://app.biorender.com/biorender-templates.

The main gene disrupted in LSF is *TP53*, a tumor suppressor gene encoding the p53 protein, fundamental for the transcription of target genes involved in cell cycle arrest, DNA repair and response to DNA damage (15). *TP53* gene is located on chromosome 17p13.1 and more than 250 different germline alterations have been reported in medical literature to date. In brain tumors, most mutations reside within the DNA binding domain, even though all the genotypic-phenotypic correlations are not fully understood (16). Despite genetic lesions in LFS have been widely studied, not all the underlying genetic defects responsible for LFS have been found. In fact, several families fulfill the definition of classical LFS without the recognition of any known *TP53* defect being found (16). Although few LFS cases have been reported with germline mutations in the *CHK2* gene, no pediatric CNS tumors have been detected in these patients, suggesting a genotype-phenotype correlation between such malignancies and *TP53* mutations (17, 18). See Figure 1 for details.

### Clinical and Therapeutic Considerations

As already mentioned, there are no clinical characteristics associated with LFS other than an increased cancer risk. Considering this and the highly penetrance of LFS, clinical and familial diagnostic criteria are essential for the diagnosis. Classic diagnostic criteria and revised Chompret criteria for LFS are reported in Supplementary Table 1 (19). It is essential to individuate families where LFS might be present as it has been demonstrated that intense tumor surveillance leads to increased survival (20).

It has been proven that *TP53* mutations are a negative prognostic factor in several tumor types, including pHGG (21). Despite the high risk of secondary malignancies after exposure to ionizing radiation, no specific treatment is available for LFS pHGG patients. Treatment strategies in these patients can be challenging, since mutations in the *TP53* gene have been associated with resistance to both chemotherapy and radiation (22). Also, LFS patients with CNS tumors show an overall worse outcome if compared to non-affected patients (22).

## Neurofibromatosis

Neurofibromatosis type 1 (NF1) (OMIM #162200), also known as von Recklinghausen disease, is a common autosomal dominant disorder with a prevalence of 1:4.000 individuals due to mutations of the *NF1* gene on chromosome 17q11.2 (23). The protein product of the NF1 gene, neurofibromin, regulates several intracellular processes, including the RAS/ERK/MAP kinase cascade and cytoskeletal assembly. Loss-of-function mutations of *NF1* gene lead to a high risk of tumor development due to decreased RAS signaling inhibition (24). Clinically, NF1 is characterized by *café au lait* macules, skin fold freckling, optic pathway gliomas, neurofibromas and plexiform neurofibromas, osseous lesions, and iris hamartomas (Lisch nodules) (23). The clinical diagnosis requires the fulfillment of at least two of the criteria as listed in Supplementary Table 2, however there are other possible manifestations that are not included in the diagnostic criteria but that can be present in patients harboring the mutation, such as macrocephaly, learning disabilities, vasculopathies and scoliosis. NF1 is associated with some CNS neoplasms in infancy, namely optic pathway gliomas and brainstem gliomas.

### Epidemiology and Cancer Spectrum

NF1 (von Recklinghausen disease) is one of the most common CPS (13). It is an autosomal dominant inherited condition and about 50% of cases are found *de novo* with no associated family history (25).

CNS neoplasms predominantly associated with NF1 are optic pathway gliomas (15–20%) and brainstem -gliomas (1–2%). Other malignant tumors can be observed in these patients such as malignant peripheral nerve sheath tumors (MPNST) and juvenile myelomonocytic leukemia (JMML) (26).

### Etiopathology

The gene involved in the pathogenesis of this syndrome is *NF1*, an onco-suppressor located on chromosome 17q11.2. The protein encoded by this gene is called Neurofibromin and is a GTPase activating protein that inhibits the product of the *RAS* oncogene, mediating the passage from GTP-RAS to GDP-RAS. RAS, in turn, is an activator of cell-cycle signaling pathways such as MAPK (RAF-MEK-ERK) and PI3K/AKT/mTOR pathways (27). *NF1* loss-of-function mutations remove this inhibition on RAS and the downstream pathways, leading to abnormal cell proliferation and tumorigenesis.

### Clinical and Therapeutic Considerations

NF1 brain tumors are considered more indolent than same histology counterparts observed in patients without NF1, and can even regress over time without treatment (28). Histologically, most of them are low-grade gliomas (LGG), with a smaller representation of pHGG (81). It is notable that NF1 associated pHGG exhibit the same genetic alterations found in sporadic pHGG (such as *P53* and *CDKN2A* alterations) (29). On the other hand, NF1 alterations are frequently found as somatic genetic lesions in sporadic HGGs of childhood (30).

Apart from LGG, differential diagnosis of pHGG in NF1 children has to include the frequent finding of Focal Areas of Signal Intensity (FASI) in these patients. These are benign lesions, usually multiple and radiologically characterized as non-enhancing, small areas without mass effect or edema. They can be found in around 70% of NF1 pediatric cases and must be differentiated from gliomas (31).

Being pHGGs very uncommon in NF1, surveillance neuroimaging is controversial and not universally recommended (24). Regardless, families should be instructed to recognize the warning signs of brain tumors.

Treatment of pHGG in NF1 is similar to sporadic cases, some reports suggest that prognosis might be better than sporadic pHGG (32, 33). As for target specific therapies, MEK inhibitors have shown promising results in NF1 patients with low grade gliomas, this result may pose the basis for future treatment strategies also in NF1-pHGG (34).

Radiotherapy is generally part of the treatment protocol, despite increased complications, namely secondary malignancies and stroke (35).

## Constitutional Mismatch-Repair Deficiency Syndrome

Constitutional mismatch repair deficiency (CMMRD) syndrome (OMIM #276300) is a childhood autosomal recessive cancer predisposition syndrome caused by a biallelic germline mutation in the DNA mismatch repair (MMR) genes, namely *mutL homolog1* (*MLH1), mutS homolog1* (*MSH2), pms2 c-terminal like pseudogene* (*PMS2)*, or *mutS homolog6* (*MSH6)* (36). Patients with monoallelic mutations in the MMR genes develop hereditary non-polyposis colorectal carcinoma (HNPCC), also known as Lynch syndrome, an autosomal dominant genetic disorder associated with increased risk of colorectal cancer, endometrial carcinoma, and other gastrointestinal and genitourinary malignancies in the fourth and fifth decades of life (37).

### Epidemiology and Cancer Spectrum

CMMRD is a rare disease with roughly 200 cases reported to date (38, 39). However, its prevalence might be underestimated and a consistent number of cases might go undiagnosed in South Asian and Middle Eastern countries where consanguinity is more prevalent (40). In CMMRD, the tumor spectrum is very broad including CNS (glioblastoma, oligodendroglioma, low-grade glioma, medulloblastoma, and other embryonal tumors), hematological, genitourinary and intestinal tract tumors (41). Among brain tumors, malignant gliomas are the most frequent CMMRD-associated tumors, typically presenting within the first 2 decades of life and accounting for 25–40% of CMMRD cancers (41). Overall, there is a high degree of consanguinity within the family, indicating that inbreeding is a major risk factor for this otherwise rare disorder.

### Etiopathology

**Figure 2:**
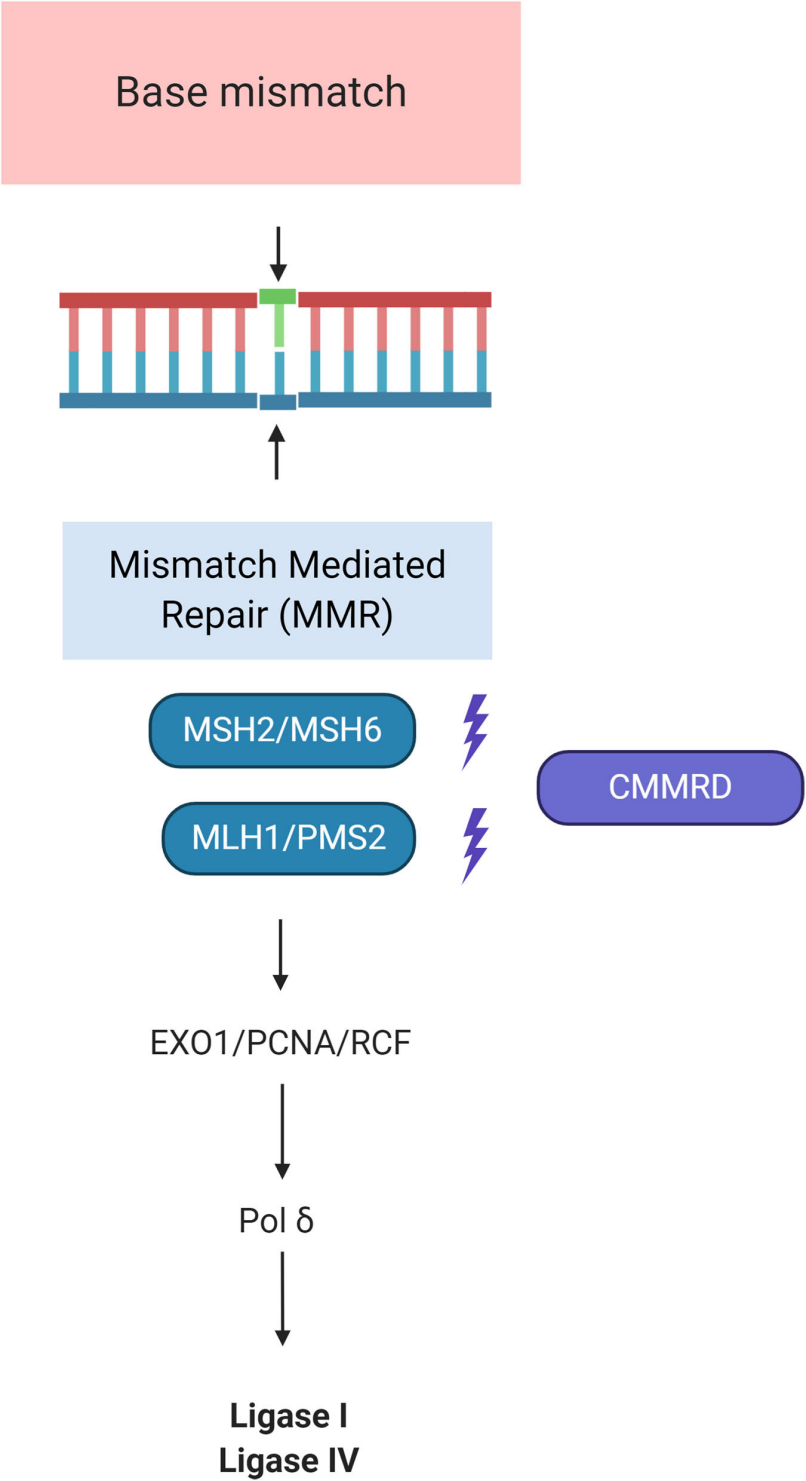
Molecular pathways of Constitutional Mismatch Repair Deficiency Syndrome (CMMRD). MSH2 dimerizes with MSH6 to form the MutSα complex, which is involved in base mismatch repair and short insertion/deletion loops. The formation of the MSH2-MSH6 heterodimer accommodates a second heterodimer of MLH1 and PMS2. This protein complex formed between the 2 sets of heterodimers enables initiation of repair of the mismatch defect by recruiting PCN/EXO1/RCF. RFC is essential for PCNA loading and function in DNA replication. PCNA loads onto double-strand breaks and promotes Exo1 damage association through direct interaction with Exo1. By tethering Exo1 to the DNA substrate, PCNA confers processivity to Exo1 in resection. This role of PCNA in DNA resection is analogous to its function in DNA replication where PCNA serves as a processivity co-factor for DNA polymerases such as polymerases δ. DNA Pol δ is an enzyme used for both leading and lagging strand synthesis by engaging Ligase I and IV. Adapted from “DNA Repair Mechanisms by BioRender.com (2020). Available online at: https://app.biorender.com/biorender-templates.

*MSH2, MSH6, MLH1*, and *PMS2* genes are involved in the mismatch repair mechanisms, one of the most important DNA repair machinery of the cell (36). Its main role is to correct errors arising during DNA replication, thus tumors arising in the context of CMMRD exhibit an extraordinary number of DNA mutations. The most common type of defects found in these “hypermutated cancers” are point mutations (single nucleotide variations) and microsatellite instability (MSI) where repetitive sequences (microsatellites) are not adequately repaired. Recently, new genetic alterations affecting this machinery have been described, such as *MSH3* variants (42), deletions of the *EPCAM* gene (43), and mutations in DNA polymerases epsilon and delta 1 (*POLE, POLD1*) (44). See Figure 2 for details.

### Clinical and Therapeutic Considerations

In addition to cancer, CMMRD patients frequently have other physical features such as cutaneous *café-au-lait* spots and hypo- or hyperpigmented spots that may mimic some of the skin features usually observed in NF1. Also neurofibromas, Lisch nodules and freckling have been reported, although less frequently than in NF1 (39, 45). Other findings have occasionally been described in these patients such as vascular anomalies, pilomatrixomas, agenesis of the corpus callosum (46), and decreased levels of immunoglobulins IgG2/4 and IgA (39). However, none of these features are mandatory to diagnose the syndrome. The penetrance of the disease is very high, reaching more than 90% by the first two decades of life. Most patients will have childhood cancer and more than one tumor, often presenting synchronously (13).

Initial screenings can be performed by immunohistochemistry showing loss of MMR protein both in normal and malignant cells. Diagnosis can be confirmed by genetic testing for the presence of biallelic mutations in one of the four MMR genes. Evidence of low grade glial lesions and premalignant, dysplastic polyps advocates for surveillance protocols to intercept asymptomatic tumors at early stages, when they are more amenable to complete resection (47). Current protocols suggest annual whole-body MRI (WBMRI) from the age of 6 years. In addition, it is recommended to start colon surveillance by colonoscopy from 6 years of age. Treatment of CMMRD tumors is complicated by resistance to standard therapies for pHGG such as temozolomide, since it requires adequate mismatch repair to perform its action.

Interestingly, immunotherapy has proved to be a promising strategy in these tumors. One of the main mechanisms through which tumors escape immune recognition and induce immunosuppression is PD-L1 overexpression of cancers that acts as a binding site for PD1. The binding of PD1 to PDL1 activates PD1 signaling that inhibits T cells allowing the tumor to evade immune attack (48). These principles have been used to develop drugs named checkpoint inhibitors that counteract the interactions of the PD1 protein. It has been demonstrated that CMMRD tumors are more responsive to PD1 blockers than MMR proficient tumors. In particular, in children with CMMRD with recurrent glioblastoma, shrinking of tumors was observed on MRI, suggesting these tumors as ideal candidates for such therapies (49).

## Ollier Disease and Maffucci Syndrome

Ollier disease (OD, OMIM 166000) and Maffucci syndrome (MS, OMIM 6145692) are related conditions characterized by multiple endochondromas and caused by somatic mutations in the *IDH1* and *IDH2* genes, respectively (50, 51). The main difference between the two conditions is the presence of hemangiomata in MS, moreover, while OD presents with multiple enchondromas, typically unilateral in distribution with a predilection for the appendicular skeleton, MS is often characterized by multiple enchondromas bilaterally distributed (51).

### Epidemiology and Cancer Spectrum

Most cases of OD and MS have been reported as sporadic, with an estimated prevalence of 1 out of 100,000 individuals, although the description of few familial cases of OD suggests a possible autosomal dominant pattern of inheritance (51). About half of the individuals with OD or MS develop a malignancy, such as chondrosarcoma (with a prevalence of 30% in both conditions), glioma, and ovarian juvenile granulosa cell tumor, accompanied by other clinical features, such as multiple swellings on the extremity, deformity around the joints, limitations in joint mobility, scoliosis, bone shortening, leg-length discrepancy, gait disturbances, pain, loss of function, and pathological fractures (51).

### Etiopathology

Mutations in the *IDH1* or *IDH2* genes have been detected in a large number of adult diffuse grade II and grade III gliomas; such high frequency has suggested a possible role for those variants as the earliest oncogenic event in these malignancies (52). It has been proven that pathogenic variants in these two genes cause an abnormal production of 2-hydroxyglutarate (2-HG), a structural analog of alpha-ketoglutarate, a key intermediate of the Krebs cycle. 2-HG competitively inhibits the active sites of multiple alpha-ketoglutarate enzymes, resulting in hypermethylation of histones and DNA, altered cell differentiation, and activation of a series of downstream enzymes (53, 54). Some of these enzymes are involved in the degradation of HIF-1 (hypoxia-induced factor 1), a key player in the cellular adaptation to low oxygen and nutrient-deprived environment and in the progression to malignancy in human solid cancers, and in the overexpression of platelet-derived growth factor receptor A (PDGFRA), implicated in the pathogenesis of leukemias, lymphomas, gastrointestinal stromal tumors, and various types of brain tumors (53–55).

### Clinical and Therapeutic Considerations

The clinical management of individuals with OD and MS is mostly focused on treating via surgery the complications arising from the enchondromas, such as fractures, growth defects, and tumors. The prevailing strategy aims to treat and remove any extraneous bone tissue preserving the limb function (51). Although gliomas are not the most frequent types of malignancies reported in OD and MS, imaging surveillance is recommended. The gliomas described in these conditions are similar to the ones caused by sporadic variants in *IDH1* or *IDH2* for their frequent location in the frontal lobe and their prevalent histological type: more commonly diffuse low-grade or anaplastic gliomas than glioblastomas (53). However, they present some substantial differences as compared to the sporadic forms: they are diagnosed at an earlier age and involve more frequently the brainstem, hinting toward an earlier origin of gliomas associated with enchondromatosis.

## Other Syndromes and pHGG

Some less-known syndromes have been associated with pHGG with lower frequency than the afore-mentioned syndromes.

One of those is the Familial Melanoma Astrocytoma Syndrome (56, 57). It is caused by germline inactivating deletion of the *CDKN2A* tumor suppressor gene. Affected individuals have a predisposition to develop melanoma and CNS tumors, most commonly astrocytoma.

Since familial predisposition to glioma has been consistently observed within non-syndromic families, an international consortium named GLIOGENE was formed in order to collect such non-syndromic glioma families, and possibly identify new genes involved in the pathogenesis of these tumors. One of the genomic regions identified by the consortium lies in chromosome 17q. According to these linkage studies the *MYO19* and *KIF18B* genes and rare variants in *SPAG9* and *RUNDC1* have been identified as candidates worthy of further investigation (58). Also, whole exome sequencing allowed the identification of mutations in *POT1* (p.G95C, p.E450X), a member of the telomere shelterin complex (59). These new findings may not only have a leading role in identifying new pathogenic pathways in gliomas but may also contribute to improve targeted treatment of this disease.

Mutations in *BRCA1* and *BRCA2*, tumor suppressor genes involved in DNA repair, have been traditionally associated with an increased risk of breast and ovarian cancer. More recently, they have been recognized to also play a role in CNS tumors (60). In particular, germline variants of *BRCA2* which is also essential for normal neurogenesis (61) have been described in individuals with brain tumors including glial tumors, meningioma and medulloblastoma (62–64).

There have been some anecdotal reports of pHGG in other syndromes (65), such as tuberous sclerosis (66), Beckwith-Wiedemann and Fanconi Anemia (67). However, these case reports do not prove a real increased risk for pHGG.

## Molecular Diagnostics of CPSs

Genetic testing in pediatric oncology is of great interest for the investigation into potentially underlying CPSs. Molecular diagnosis of a CPS can influence cancer surveillance program initiation or frequency, and directly impact treatment decisions. Genetic diagnostic laboratories have introduced next-generation sequencing (NGS) technologies into their practices. NGS has specific advantages over traditional Sanger sequencing, considered the gold standard for mutation analysis for many years, as multiple genes in several patients can be tested simultaneously. Different approaches are being used, and currently, most laboratories that use these technologies are performing targeted gene panel testing or clinical whole exome sequencing (WES), more rarely whole genome sequencing (WGS). These revolutionary technological advances have drastically reduced sequencing costs and shortened the turnaround time, increasing the detection rate (68). Multi-gene panels usually include high and moderate penetrance genes, and sometimes, some low or unknown risk genes, that offer the advantage of identifying germline pathogenic variants in genes that would not normally be tested based on the patient's diagnosis (69). Unfortunately, depending on the disease, between 70 and 92% of the patients remain mutation-negative or undiagnosed after gene-panel testing (70). It is possible that variants in genes not included in these panels contribute to the cancer risk and WES or WGS can explore the genetic basis of familial syndromes in a more extensive way, permitting to identify new high- and moderate-risk cancer predisposition genes. WES of parent-child trios has become a widely used strategy to identify presumably pathogenetic genetic variants in children with rare diseases. However, it has not yet been routinely implemented in pediatric oncology, with few exceptions (71). Genome-wide approaches generate huge amounts of genetic data and it remains challenging to interpret the identified variants. Such data interpretation needs close collaboration among bioinformaticians, molecular geneticists and clinicians. However, as sequencing costs are decreasing and computer and technological resources are expanding, genome-wide analysis will become more common in the clinical practice and hopefully help to advance on the path of personalized medicine, by providing more precise genetic diagnoses and better molecular information for more effective treatments.

## DNA Methylation Profiling

Recently, a machine learning approach for classification of CNS tumors based on the analysis of global DNA methylation profiling has been developed and introduced to reach a histopathological-molecular integrated diagnosis, discriminating tumor classes and ameliorating diagnostic precision (72, 73). In detail, the developed “Classifer” provides a methylation-based classification assigning a subgroup score for an index tumor compared to 91 different brain tumor entities. Furthermore, it also provides a chromosomal copy-number variation (CNV) analysis.

Interestingly, Capper and colleagues found that a high proportion of unclassifiable CNS tumors were associated with various hereditary tumor syndromes, and/or diagnosed in childhood (73). Additional chromothripsis and unusual complex chromosomal changes should also be considered as a cue for Li–Fraumeni syndrome-associated tumors.

## Conclusions

Pediatric HGG cancer predisposition syndromes are rare and diverse pathological conditions that may be present in children with CNS tumors and deserve consideration.

Knowing when to suspect one of these predisposing syndromes is essential for the pediatric oncologist, not only to make the correct diagnosis, but also to formulate a more accurate prognostic judgment and provide an adequate treatment. Moreover, it is mandatory to refer the family for genetic counseling when such conditions are suspected. This latter aspect is of particular relevance since it has been demonstrated that close surveillance can decrease the morbidity and mortality in these patients.

The ever-growing knowledge of the genetic mechanisms underlying cancer is a key tool in the understanding of this disease, opening new scenarios for the introduction of molecular target therapy.

Since these conditions are extremely rare, several patients' associations have been created to help families find the nearest structure for follow-up and to raise funds and consciousness for these diseases.

## Author Contributions

GC, GD, AM, and AC designed the study. GC, GD, MV, LB, RC, and EM cured the literature research and its organization. GC, EA, FD, and MR cured the literature research focusing on the genetics aspect. GC, GD, EM, and LB wrote the final version of the manuscript. AM, AC, FL, and LB critically revised the manuscript for intellectual content. Finally, all authors approved the version to be published and agreed to be accountable for all aspects of the work in ensuring that questions related to the accuracy or integrity of any part of the work are appropriately investigated and resolved.

## Conflict of Interest

The authors declare that the research was conducted in the absence of any commercial or financial relationships that could be construed as a potential conflict of interest.
