# Supplementary material for: Cancer Predisposition Syndromes Associated With Pediatric High-Grade Gliomas
Source: Front Pediatr. 2020 Nov 12;8:561487. doi: 10.3389/fped.2020.561487 (PMC7690624; doi:10.3389/fped.2020.561487)
Supplement: Supplementary file 1 [file Table_1.docx]

**Supplementary Materials**

**Table 1.** Classic diagnostic criteria and revised Chompret criteria for LFS1

| **Classical Li-Fraumeni syndrome (LFS) criteria: All obligatory**  **Li et al.** | A proband with Sarcoma diagnosed under the age of 45 years  AND  A first degree relative with any cancer under 45 years  AND  Another first or second degree relative with either cancer under 45 years or a sarcoma at any age |
| --- | --- |
| **LFS Chompret criteria revised: only one obligatory**  **Tinat et al.** | Family History: A proband with a tumor belonging to the LFS tumour spectrum (soft tissue sarcoma, osteosarcoma, brain tumors, pre-menopausal breast cancer, adrenal cortical carcinoma, leukaemia, lung bronchoalveolar cancer) before 46 years AND At least one first or second degree relative with an LFS tumor (except breast cancer if the proband is affected by breast cancer) before 56 years or multiple primary tumors  OR  Multiple primitive tumors: A proband with multiple primary tumors (except multiple breast tumors), two of which belong to the LFS tumor spectrum and the first of which occurred before 46 years  OR  Rare tumors: A proband with adrenal cortical carcinoma or choroid plexus tumor, irrespective of the family history  OR  Early-onset breast cancer: breast cancer before age 31 years |

**Table 2.** Diagnostic criteria for NF1: Fulfillment of at least 2 for diagnosis (modified from (14))

| Six or more cafe-au-lait macules >5 mm in diameter in prepubertal and >15 mm in diameter in postpubertal individuals |
| --- |
| Two or more neurofibromas of any type or one plexiform neurofibroma |
| Freckling in the axillary or inguinal regions |
| Optic glioma |
| Two or more Lisch nodules (iris hamartomas) |
| A distinctive bony lesion, such as sphenoid dysplasia or thickening of the long bone cortex with or without pseudoarthrosis |
| A first-degree relative with NF1 based upon the above criteria |
